# EXPERTS 1—experiences of long-term life-limiting conditions among patients and carers: protocol for a qualitative meta-synthesis and conceptual modelling study

**DOI:** 10.1136/bmjopen-2014-007372

**Published:** 2015-04-02

**Authors:** Carl R May, Jayne Masters, Lindsay Welch, Katherine Hunt, Catherine Pope, Michelle Myall, Peter Griffiths, Paul Roderick, Julie Glanville, Alison Richardson

**Affiliations:** 1 Faculty of Health Sciences, University of Southampton, Southampton, UK; 2 NIHR CLAHRC Wessex, University of Southampton, Southampton, UK; 3 University Hospital Southampton NHS Foundation Trust, Southampton, UK; 4 Solent NHS Trust, Southampton, UK; 5 Faculty of Medicine, University of Southampton, Southampton, UK; 6 York Health Economics Consortium, University of York, York, UK

**Keywords:** chronic disease, multimorbidity, patient complexity, treatment burden, workload

## Abstract

**Introduction:**

Increasing numbers of the population are living with long-term life-limiting conditions with a significant proportion characterised by multimorbidity. Patients with these conditions often experience high volumes of clinical interaction involving them, their caregivers and healthcare providers in complex patterns of organising, coordinating, negotiating and managing care. A better understanding of the sources of experienced complexity and multimorbidity, from the patient perspective is paramount to improve capacity and manage workload to promote improved experience of illness, more effective healthcare utilisation and improved healthcare outcomes. To better understand the sources of complexity we will undertake an evidence synthesis of qualitative studies of patient and informal carer experiences of three common long-term life-limiting conditions. We will investigate what is known about these diseases at different stages in disease progression, treatment regimens and places of care.

**Method and analysis:**

We will include qualitative studies of patients’ and carers’ (aged >18) accounts of their experiences of healthcare provision in a range of settings and healthcare systems. We will conduct an extensive electronic database search of publications in English between 2000 and 2014. Results and discussions sections of the papers will be regarded as formal data using the constant comparison method of qualitative analysis. From the meta-synthesis results, we will build a conceptual model of mechanisms and processes that shape patients’ journeys towards end of life to suggest where in the patient journey new interventions to improve patient and carer experience can be developed and delivered. The study is being conducted between 1 December 2014 and 31 December 2015.

**Ethics and dissemination:**

No human subjects or personal data are involved and no ethical issues are anticipated. An important element of dissemination is informing user communities about the practical implications of the work through workshops, meetings and social media. Scientific results will be published in peer reviewed journals and disseminated through conferences.

**Trial registration number:**

PROSPERO CRD42014014547.

**Strengths and limitations of this study**

- Comprehensive search strategy.
- Focus is on patient and carer experience of illness trajectories rather than specific aspects of index conditions or care processes which enable a better understanding of patient journeys through care.
- The work will help to make better sense of patient experiences of multimorbidity to prioritise from the patient and carer perspective ways to improve capacity and better manage workload to promote improved experience of illness.
- No limitations of the systematic review and meta-synthesis are anticipated.

## Introduction

The epidemiological shift from communicable and acute to non-communicable chronic diseases over the last century, and the demographic transition from younger to older populations, has created policy and practice problems that healthcare providers are now confronting and represent issues of clear and immediate importance.

First, an increasing proportion of the population are older (age >65); that this group is characterised by long-term conditions that have life-limiting consequences; that a significant proportion of this group are characterised by multimorbidity (defined as the coexistence of two or more long-term life-limiting conditions in an individual), and that those conditions appear earlier (perhaps up to 20 years earlier) in populations exposed to multiple socioeconomic disadvantages. For patients with conditions such as chronic heart failure, chronic kidney disease and chronic obstructive pulmonary disease (COPD), these epidemiological factors define important aspects of need for, and demands on, services.^1–3^

Second, patient experiences of multiple comorbid long-term life-limiting conditions are characterised by cycles of hospital admission and discharge, supported self-care at home and readmission as acute episodes of disease and exacerbations of symptoms occur over time. Cyclic patient careers and multiple exacerbation events are thus very visible elements of the increasingly complex trajectories experienced by many patients and family caregivers. These admission-discharge-readmission cycles are common but involve patients, their significant others and healthcare providers in what are sometimes very complex experiences of negotiating and organising care. Healthcare providers seek to control these cycles, by reducing hospital admissions and readmissions, emergency department visits and increasing management in ambulatory or primary care.^4–8^

Third, an important element of complexity for these patients (and their significant others) is the necessity to work across a spectrum of activities that can range between experiences of formal healthcare (clinical interventions, diagnostic procedures, therapeutic regimens and fundamental essential care delivered by health professionals) and informal healthcare/self-care (self-monitoring, symptom and medication management, help-seeking and other health-related activities) within the context of complex social networks and personal lives. Healthcare activities involve participants in a wide range of tasks and relationships that are, in turn, characterised by different kinds of organisational accountabilities and personal obligations.^9–12^

The array of clinical, personal and relational factors that shape experiences of interactions with healthcare services and providers are complex and this is compounded by the diversity of organisational contexts in which they are located.^13^ This makes patient experiences and trajectories difficult to trace and conceptualise.^14^ However, we have already made important conceptual advances in three relevant areas. First, we have developed a strong theory of the implementation and integration of clinical interventions, treatment regimens and other health technologies by individuals in formal clinical settings and in the home.^15^ ^16^ Second, drawing on this theoretical model, a methodology of care provision—*minimally disruptive medicine*^17^—was developed that emphasises the importance of the simplifying the organisation and delivery of care to reduce treatment burden, improve treatment adherence and improve healthcare outcomes.^18^ ^19^ Third, we have developed a strong theory of patients’ relational networks capacity to take on self-care and healthcare tasks.^20^ ^21^

Existing literature in the field has focused on complexity as a problem of healthcare systems,^13^ ^14^ ^22^ on relational aspects of patients’ lives,^23^ ^24^ and on experiences of chronic illness as a source of transformations in self-perception and personal relations.^25–27^ Patient focused qualitative studies and systematic reviews have tended to focus on index conditions, for example, heart failure^28^ or stroke.^18^ These studies and reviews are important and valuable, but we need to extend this work to develop an analysis of patient trajectories or careers through healthcare services so that we can understand patient journeys through care, and consider the ways that underpinning mechanisms and processes affect each other and shape the whole, to inform the redesign of healthcare provision so that it is better coordinated and more patient centred in its delivery. Most reviews have focused on specific aspects of index conditions or care processes. This review will focus on patient and carer experiences of illness trajectories. This will help us to make better sense of patient experiences of multimorbidity;^29^ ^30^ to prioritise from the patient and informal carer perspective ways to improve capacity and manage workload in order to promote better experience of illness; and to link this to experiences of socioeconomic and demographic advantage and disadvantage. Our aim is to support the development of more effective mechanisms for healthcare utilisation, and improved healthcare outcomes. Further, we need to understand the role that social structural factors (socioeconomic advantage, gender, ethnicity and age) play into these experiences.

To better understand the sources (and thus potential solutions) of experienced complexity, we will undertake an evidence synthesis of qualitative studies of patient and informal carer experiences of three conditions: chronic heart failure, chronic obstructive pulmonary disease, and chronic kidney disease. These conditions are common ones that place significant burdens of both symptoms and treatment on sufferers and lead to significant demands on health services. Our evidence synthesis will investigate what is known about experiences of these diseases at different waypoints in disease progression, treatment regimens, and place of care. By these means we will identify, characterise and explain (1) the general features of complex relational and organisational processes that shape patient and carer experiences of care in long-term conditions towards the end of life; and (2) the contingent, or disease/healthcare system specific features of these conditions. Making a clear distinction between general and contingent elements of patient journeys will enable us to identify those factors that matter in *specific* index conditions and healthcare systems, and those that are common to long-term life-limiting conditions across a range of healthcare systems. Identifying and understanding these will facilitate the identification of points in the patient journey at which new interventions to co-ordinate and deliver care and improve patient and informal carer experience can be developed and delivered.

## Methods (1) systematic review and meta-synthesis

### Objective

To identify, characterise and explain factors that shape patient journeys through care in three long-term life-limiting conditions (chronic heart failure, chronic obstructive pulmonary disease and chronic kidney disease), by undertaking a systematic review and qualitative meta-synthesis of studies relevant to patient and informal carer experiences of their interactions with health and social care services.

### Eligibility criteria

We will include reports that meet all of the following criteria.

- *Participants:* aged >18; diagnosed with chronic heart failure, chronic kidney disease or chronic obstructive pulmonary disease or their informal carers.
- *Reports:* of the results of qualitative studies of patients’ or carers’ accounts of their experiences of healthcare provision by health professionals in triage services, emergency departments, inpatient hospital care, outpatient/ambulatory care departments, primary care service/family practice doctor's offices, community nursing services or at home published in peer-reviewed journals
- *Study designs* including: primary studies, qualitative systematic reviews, qualitative meta-syntheses and meta-ethnographies. Qualitative analyses using data collected in studies where semistructured and unstructured interview techniques or ethnographic or similar observation techniques are used to perform all (or in the case of mixed methods studies a significant proportion of) data collection will be eligible.
- *Settings:* healthcare systems where general practice/family practice services interact with hospital/tertiary care facilities in Europe, North America and Australasia.
- *Date of publication*: between 1 January 2000 and 31 December 2014.
- English *language*.

We will exclude reports of treatment effectiveness, for example, RCTs, where the focus is on the treatment effect rather than the patient's or carer's experience; reports of healthcare organisation or delivery which are not focused on the patient's or carer's experience; reports which do not report the results of qualitative research with patients or carers; and editorials, notes, letters and case reports.

### Systematic literature searches

Literature searching will be contracted to information specialists, the systematic reviews group of the York Health Economics Consortium (YHEC). In collaboration with YHEC, we will develop a search strategy for systematic searches of the following databases: Science, Social Science and Arts and Humanities Citation Indices (Web of Science; CINAHL (EBSCO); EMBASE (Ovid); MEDLINE (Ovid); PsycINFO (Ovid); Scopus). YHEC will run searches and conduct de-duplication of citations, providing Endnote database files of citations for screening. The search strategy contains several different search approaches reflecting the fact that relevant records may not be consistently described. The draft conceptual strategy is shown below:

- (Index conditions OR generic long term conditions) AND experience terms
- (Index conditions OR generic long term conditions) AND concept of patients AND qualitative research terms
- 1 OR 2
- limit 3 to English language
- limit 4 to records including abstracts
- limit 5 to records published in the year 2000 onwards
- 6 NOT (editorials OR comments etc.)

A complementary search strategy will be developed to locate studies that pertain to informal carers.

### Screening

Screening will start with an assessment of relevance of citations and abstracts by two reviewers independently. Any studies which are eligible (meet the criteria set out above) or may be eligible (ie, where the content is unclear or reviewers disagree) will be obtained in full text. Full text papers will be screened by two reviewers independently.

### Quality assurance of included articles

There are many proposed sets or reporting criteria for qualitative studies.^31^ ^32^ National Institute for Health Research Collaboration for Leadership in Applied Health Research and Care (NIHR CLAHRC) Wessex has adopted the RATS framework^33^ to guide researchers on assessing the quality of qualitative research proposals and papers. RATS provides clear criteria for identifying high-quality reports. It does not provide a formal scoring procedure for studies. However, since there is no universally accepted reporting standard for qualitative studies RATs can only guide decision-making on eligibility for inclusion. Two members of the team will assess quality against RATs criteria. Reports that provide insufficient information about sample, question, method and setting will be excluded from the review.

### Data extraction

Data extraction will be conducted by two researchers. Where disagreements about inclusion or abstraction occur, they will be arbitrated by a third member of the team. We will develop a core data extraction instrument. We will pilot data abstraction procedures and the data abstraction instrument on a random sample of five papers.

### Data analysis

We will treat the results and discussions sections of included papers as the formal data for qualitative analysis, and follow the following set of analytic procedures. We will undertake a preliminary analysis of five purposively sampled reports, coding data along the analytic axes described below.

1. *Changing experiences over time and space*: We are interested in patients’ journeys through care, we will identify and characterise those data that tell us about how experiences of healthcare change over time and how these changes are affected by episodes of disease and disease severity; by changing relationships with different health professionals and clinical services; and by changing interactions with different healthcare providers.
2. *Experiences of healthcare practices*: We are interested in factors that shape patients’ and informal carers’ experiences of their journeys through care. We will identify and explore those data that tell us about their experiences of actual clinical care, its modes of delivery and wider patterns of organisation.
3. *Experience-based evaluations*: We are interested in patients’ and informal carers’ experiences of the factors that shape their journeys through care, we will identify and explore those data that tells about the ways that they understand these factors and evaluate their effects on their care.

On the basis of this preliminary work, we will construct a coding frame for constant comparative thematic analysis of qualitative data.^34^ We will adapt and refine this coding frame over the course of the review, as thematic analysis reveals commonalities and differences in patient accounts of their experiences of care. Coding and initial interpretation will be undertaken by three team members. Although inter-rater reliability testing is a problem in qualitative research,^35^ we will randomly sample coding activity to ensure quality control. We already have experience of developing, applying and operationalising such approaches in successful systematic reviews of qualitative studies.^36–39^ In this study, we will use NVivo software to manage qualitative data.

## Methods (2) conceptual modelling

### Objective

To build a robust generic conceptual model of the mechanisms and processes that shape patient journeys towards end of life in long-standing life-limiting disease. This will suggest types of interventions that will promote minimally disruptive healthcare and the waypoints in patient trajectories at which these interventions should be implemented.

### Conceptual modelling

The qualitative meta-synthesis will identify and characterise the ways that experiences of healthcare are structured over time; in interaction with different modes of service provision; and how they lead to different patterns of participants’ evaluation. Results will suggest factors that are common to all three index conditions and all healthcare systems that included papers reporting factors that are common to long-term conditions (core processes and mechanisms), and factors that are specific to an index condition or a specific healthcare system (contingent processes and mechanisms). We will then build a robust conceptual model that explains the operation of these processes and mechanisms. This will facilitate the identification of supportive interventions with the potential to be routinely incorporated in practice. To do this we will build on the development of two empirically grounded theoretical models relevant to the problem of negotiating and embedding processes of care (normalisation process theory,^15^ and burden of treatment theory).^21^ To achieve this, we will use directed content analysis,^40^ of qualitative data to build taxonomies of factors that can be demonstrated to shape patient and carer experiences. We will characterise these factors, showing how they are formed, how they are related to each other and how they relate to the contexts in which they operate.

Comparative approaches to these data will enable us to identify a set of factors common across contexts, (these will enable us to develop decision trees and logic models of interaction and organisational behaviours).^41^ We will also identify factors that are contingent or context specific, and integrate these into the model where they suggest robust underlying mechanisms that shape the potential for supportive interventions. The final stage of this task is to assemble the products of integrative analysis as a set of empirically grounded, but theoretically robust generative principles, to direct and structure future policy and practice interventions and to characterise the mechanisms and processes that such interventions would seek to restructure. We have considerable experience of model building on the basis of systematic reviews and meta-syntheses,^18^ ^36^ ^42–45^ and are well placed to develop a practically useful conceptual framework from which to inform interventions.

### Dissemination and implementation

We will ensure wide dissemination of this systematic review and qualitative meta-synthesis to include publication in peer reviewed open-access journals, research meetings and conference presentations. An important part of dissemination is informing user communities of patients, informal carers and professionals about the practical implications of this work. To ensure maximum integration and effectiveness of translating study outcomes into practice, key clinical academics from the team will lead on work to enhance National Health Service (NHS) capacity to apply and embed evidence to improve the quality of experience and treatment of those with long-term conditions. This will be achieved through close working, codevelopment of interventions and knowledge translation between researchers and those working in the health service. A central part of the process of implementing findings will take place through a stakeholder panel consisting of patients and carers, members of the public (including patient and public involvement (PPI) representatives and patient advocacy organisations), clinicians, commissioners and service managers. We will consult with stakeholders, using consensus methods, to develop strategies and workable plans for implementation.
